# The duration of intrauterine development influences discrimination of speech prosody in infants

**DOI:** 10.1111/desc.13110

**Published:** 2021-04-04

**Authors:** Johanna Alexopoulos, Vito Giordano, Charlotte Janda, Silvia Benavides‐Varela, Rainer Seidl, Stephan Doering, Angelika Berger, Lisa Bartha‐Doering

**Affiliations:** ^1^ Department of Psychoanalysis and Psychotherapy Medical University of Vienna Vienna Austria; ^2^ Department of Pediatrics and Adolescent Medicine Comprehensive Center for Pediatrics Medical University of Vienna Vienna Austria; ^3^ Department of Developmental Psychology and Socialization University of Padova Padova Italy

**Keywords:** fNIRS, functional imaging, language development, preterm birth, speech discrimination

## Abstract

Auditory speech discrimination is essential for normal language development. Children born preterm are at greater risk of language developmental delays. Using functional near‐infrared spectroscopy at term‐equivalent age, the present study investigated early discrimination of speech prosody in 62 neonates born between week 23 and 41 of gestational age (GA). We found a significant positive correlation between GA at birth and neural discrimination of forward versus backward speech at term‐equivalent age. Cluster analysis identified a critical threshold at around week 32 of GA, pointing out the existence of subgroups. Infants born before week 32 of GA exhibited a significantly different pattern of hemodynamic response to speech stimuli compared to infants born at or after week 32 of GA. Thus, children born before the GA of 32 weeks are especially vulnerable to early speech discrimination deficits. To support their early language development, we therefore suggest a close follow‐up and additional speech and language therapy especially in the group of children born before week 32 of GA.

## 1 INTRODUCTION

Worldwide, 15 million babies annually are born preterm, and preterm birth rates are increasing (WHO, 2020). Children born prematurely have a greater risk of developing long‐term health complications including motor, behavioral, and cognitive difficulties. A meta‐analysis involving more than 64,000 children born preterm found a strong relationship between gestational age (GA) at delivery and later cognitive abilities (Allotey et al., 2017). In this analysis, GA at birth accounted for 48% of the observed variance in performance IQ and 38% of the variance in verbal IQ at the age of 2–18 years and above.

Language developmental delays are often the first signs of cognitive deficits and are among those most commonly reported in preterm born children. Many children born preterm exhibit significant language developmental delays in vocabulary growth and grammatical skills (Arpino et al., 2010; Barre et al., 2011; Guarini et al., 2016; Taylor et al., 2013; Vohr, 2014). In primary school, preterm born children frequently demonstrate poor reading and writing acquisition (Guarini et al., 2009; Wolke et al., 2008). These early deficits often result in persistent impairments in literacy and syntax and affect later academic achievement (Guarini et al., 2010; Twilhaar et al., 2018; van Noort‐van der Spek et al., 2012).

Auditory development already begins in the fetus (Lim & Brichta, 2016; Mejdoubi et al., 2016). The last trimester of pregnancy is an important period for the development of the auditory cortex. During this period, the functional maturation of the auditory cortex is increasingly driven by environmental stimuli (Chang & Merzenich, 2003; Hepper & Shahidullah, 1994). Consistent with this, behavioral studies have shown that fetuses habituate to their mother's language and voice (DeCasper & Fifer, 1980; Moon et al., 1993), familiar melodies (Hepper, 1988), or stories heard during pregnancy (DeCasper & Spence, 1986). These formed memories for recurrent external stimuli, known as neural memory traces, are a prerequisite for successful speech perception and discrimination (Bartha‐Doering et al., 2015; Graven & Browne, 2008) and enable neonates to generate specific learned behaviors (Partanen et al., 2013). The newborn's cry, for example, is shaped by their native prosody (Mampe et al., 2009). Accordingly, auditory discrimination abilities can be verified from the first days of life (Kujala et al., 2004; Mahmoudzadeh et al., 2013; Nazzi et al., 1998; Ramus et al., 1999), and prenatal auditory experiences have been shown to have a significant influence on the accuracy of the brain's auditory discrimination (Partanen et al., 2013). These abilities may support language acquisition during infancy: studies in full‐term infants have shown an association of early auditory discrimination abilities and later language skills (Benasich & Tallal, 2002; Kuhl & Rivera‐Gaxiola, 2008); early phonological discrimination is related to later literacy skills (Schaadt et al., 2015; van Zuijen et al., 2013), and early prosodic discrimination predicts vocabulary growth (Cristia & Seidl, 2011).

Preterm birth interrupts both structural and functional auditory cortex development (Chang & Merzenich, 2003; Harshaw & Lickliter, 2011; Monson et al., 2018). Before the brain has reached full‐term maturity the auditory cortex has shown to be more adaptive to maternal sounds than environmental noise (Webb et al., 2015). Yet, the early postnatal hearing experience in preterm infants differs significantly from the intrauterine hearing experience of fetuses of the same GA. Very recently, we measured postnatal hearing exposure of preterm infants inside the incubator at neonatal wards (Bertsch et al., 2020). With this study, we demonstrated that preterm born children nursed within an incubator are exposed to high levels of noise, caused by air supply systems, which hinder them from perceiving speech sounds from outside the incubator. Both the high noise levels and the deprivation of early speech perception may be reasons for reduced auditory speech discrimination in preterm infants already at term‐equivalent age (Bartha‐Doering et al., 2019). In the consequence, preterm infants often display deficits in phonological and prosodic discrimination during early and middle childhood (Herold et al., 2008; Peña, Pittaluga, & Farkas, 2010; Peña, Pittaluga, & Mehler, 2010).

**Research Highlights**

- Functional near‐infrared spectroscopy was used to study neural speech discrimination in 62 neonates born between week 23 and 41 of gestational age (GA).
- We found a significant positive correlation between GA at birth and neural speech discrimination at term‐equivalent age.
- We furthermore identified a critical threshold of intrauterine auditory cortex development around week 32 of GA.
- This study underlines the importance of close follow‐ups and early speech and language therapy especially in children born before week 32 of GA.

Previous research thus highlights the importance of the fetal development of the auditory cortex in utero as the basis for subsequent language development. The course of intrauterine auditory cortex development, which may be disrupted by preterm birth, is yet unknown, and research on the association of GA at birth and auditory discrimination abilities at term‐equivalent age is not available. We thus do not know whether there is a gradual intrauterine development of neural auditory memory traces, and/or whether there exists a critical threshold by which the auditory cortex is sufficiently developed to adequately discriminate speech sounds. Critical thresholds for preterm delivery have already been described, for example, for brain maturation and long‐term morbidity (e.g., Davidesko et al., 2020; Wu et al., 2017), it may thus be hypothesized that such a critical threshold also exists for auditory cortex development in utero.

Information about the relationship between the duration of intrauterine development and auditory speech discrimination at term‐equivalent age would not only shed light on the neural architecture of very early language development, but would also help clinically in identifying infants at particular risk for later language deficits at a very early stage of development. This could improve planning of early therapy strategies, as early intervention of auditory language discrimination possibilities increases the chance to ameliorate language developmental delays (Guzzetta et al., 2011). In preterm birth, treatment could start within the first months of age, when plasticity of the brain is thought to be greatest (Fiori & Guzzetta, 2015; Martinez‐Biarge et al., 2010). The present study therefore investigated a large sample of infants born between week 23 and 41 of GA with a prosodic speech discrimination paradigm at term‐equivalent age. We used functional near‐infrared spectroscopy (fNIRS), which enabled us to measure localized brain responses of neonates lying in their cribs within the neonatal ward. With this study, we aimed at investigating the possible link between the duration of intrauterine development and auditory speech discrimination at term‐equivalent age.

## 2 METHODS

### 2.1 Participants

Between 2015 and 2019, 78 neonates born between week 23 and 41 of GA were prospectively enrolled at the Division of Neonatology at the Medical University of Vienna. Inclusion criteria were (1) normal auditory evaluation as measured by auditory brainstem response; (2) normal neurological findings including normal clinical examination and normal head ultrasound scan; (3) both parents native speakers of German; and (4) normal language and reading development in both parents. Exclusion criteria were chromosomal or congenital anomalies. The study was conducted in accordance with the Declaration of Helsinki (1973, revised in 1983) and approved by the Ethics Committee of the Medical University of Vienna. Written informed consent was obtained prior to the experiment from one parent in all children.

All infants were investigated between week 36 and 42 of GA. After fNIRS measurements, 16 participants had to be excluded from further analysis due to excessive motion artifacts and/or movement of the probes, resulting in a total of 62 neonates (33 females) presented in the upcoming analysis. Clinical data of the infants included in the analyses are shown in Table 1. We furthermore calculated the difference between the individual birth weight and the population's average weight for this specific GA at birth, taken from fetal growth charts for estimated fetal weight (Kiserud et al., 2017). While GA at birth significantly correlated with birth weight (*r* = 0.950; *p* < 0.001), it was not associated with the individual deviation from age appropriate weight at birth (*r* = 0.022, *p* = 0.867).

**Table 1:** Clinical data of study participants (*n* = 62)

	Mean/median^a^	SD	Range
At birth			
GA at birth (weeks)	32.64	5.29	23.57–41.29
Head circumference at birth (mm)	29.63	4.66	21.00–38.00
Birth weight (g)	1906.70	1058.80	570.00–3990.00
Birth length (mm)	42.31	7.55	28.00–56.00
Apgar score at 1 min	8		6–10
Apgar score at 5 min	9		8–10
Apgar score at 10 min	9		9–10
At examination			
GA at examination (weeks)	38.44	1.60	36.00–42.00
Head circumference at examination (mm)	33.09	1.87	29.00–38.00
Weight at examination (g)	2691.25	595.99	990.00–3990.00
Length at examination (mm)	47.08	3.74	36.00–56.00

^a^ Mean is given in metric data, median in ordinal data.

Abbreviation: GA, gestational age.

### 2.2 fNIRS paradigm

A well‐known speech discrimination paradigm was used. This paradigm had proven speech discrimination abilities in full‐term born neonates and had shown robust activations in temporal and frontal brain areas (Bartha‐Doering et al., 2019; Dehaene‐Lambertz et al., 2002; Peña et al., 2003; Sato et al., 2012).

Auditory stimuli consisted of speech samples collected by recording a female speaker reciting a children's story using infant‐direct speech (Lobe & Weigel, 1972). The speech samples were edited into 15 s sequences with well‐formed prosodic units and each sequence was then reversed, resulting in a total of 10 native (forward) speech stimuli and 10 backward speech stimuli with the same phonetic features, but with distorted semantic and prosodic information. The presentation of backward and forward speech stimuli was counterbalanced, and each sequence was followed by silence with randomized length (15–30 s). The total stimulation was 600–900 s or a maximum of 15 min. A detailed description of the fNIRS paradigm was given previously (Bartha‐Doering et al., 2019).

### 2.3 fNIRS data acquisition

Data were recorded using the ETG‐4000 optical topography system (Hitachi Medical Corporation, Japan) with 10 fibers for emission and 8 fibers for detection, resulting in a total of 24 channels. The separation between emitters and detectors was 2 mm. The laser diodes emitted near infrared light at two different wavelengths, 695 and 830 nm, respectively, and total laser power was set at 0.75 mW. After the light was transmitted by the optical fibers to the head, the detector fiber bundle guided back the remaining light to the optical topography system with a sampling rate of 0.1–10 Hz. The optical fibers were embedded in soft silicon cushions of two light‐weight probes designed for use with neonates (Hitachi Neonate Probes). These probes were placed directly above the ear using the bilateral preauricular points as the reference to align the bottom finger of the probe (channels 3, 6, 8, and 11 in the left hemisphere; channels 17, 19, 22, and 24 in the right hemisphere) with the temporal areas (T3–T5 and T4–T6 lines in the left and right hemispheres, respectively).

Infants were tested in a quiet, dimly lit room within the neonatal ward lying in their cribs in a state of rest or sleep. The position of the head was supported with a gauze diaper to ensure a straight posture of head and neck. One parent attended the measurement. The stimuli were presented using two loudspeakers positioned at a distance of approximately 2 m in front of the baby and an angle of 30° from the infant's head.

### 2.4 Data processing and analyses

FNIRS data were pre‐processed using open source software HOMER2 on MATLAB (R2013b, Mathworks, Natick, MA, USA) (Huppert et al., 2009). First, raw optical intensity data series (voltage) were converted into changes in optical density data. Then, channels with very high or very low optical density and channels with low signal to noise ratio were pruned from individual participants’ datasets and epochs with tremendous movement artifacts. Motion artifacts were corrected using targeted principal component analysis (PCA), where PCA was applied only on segments of data identified as motion artifacts (Yucel et al., 2014). This method should avoid over‐correction of signals to overcome the problem of removing desired signals. Components accounting for 95% of covariance of data were filtered out. Remaining artifact segments that could not be corrected were automatically identified and rejected. On average, we excluded 2.31 (+/−1.89) forward sequences and 1.98 (+/−1.46) backward sequences. There was no significant difference in the amount of sequences excluded between the two conditions (*t* = 1.680; *p* = 0.098). Next, high‐frequency instrument noise in optical density data were eliminated using a low‐pass filter with a cut‐off frequency of 0.5 Hz. Baseline drifts and pulsation due to heartbeats were eliminated using a high‐pass filter of 0.01 Hz. Changes in the concentration of oxyhemoglobin (HbO) and deoxyhemoglobin (HbR) were calculated from changes in optical density using the modified Beer–Lambert law with a partial path‐length factor for both wavelengths of 6.0. Since HbO is supposed to be the strongest indicator for neural responses in the neonatal fNIRS, further analyses were focused especially on HbO signal changes (Gervain et al., 2011; Lloyd‐Fox et al., 2010). Analysis of HbR concentration changes can be found in the supplementary material.

### 2.5 Data analysis

For each individual participant, changes within the defined source‐detector channels were exported by averaging all blocks for each condition starting 5 s pre‐stimulus onsets until 30 s post‐onset. The 30 s epochs post stimulus onset were aligned to the 5 s baseline preceding the presentation of the stimulus. Based on visual inspection, mean HbO concentrating changes between 2 and 20 s post stimulus‐onset were subjected to further analysis. For further analysis, neural speech discrimination was assessed by calculating the difference between mean HbO changes following forward speech stimuli and backward speech stimuli including all time points and all channels across both hemispheres.

To investigate the impact of GA at birth on neural speech discrimination at term‐equivalent age, data analysis approach followed a three step procedure: (1) a correlation analysis, to investigate linear changes across the whole group, (2) a hierarchical cluster analysis to identify possible subgroups within the whole sample, and (3) a cluster‐based permutation test to investigate differences between the two speech conditions over the whole time interval of 30 s post‐stimulus and each individual channel within each subgroup.

Finally, to analyze specific differences between obtained clusters and groups of neonates, respectively, a cluster‐based permutation analysis taking into account each channel and sampling point was applied.

#### 2.5.1 Correlation analysis within the whole sample

To assess the relation between GA at birth and speech discrimination abilities at term‐equivalent age, the individual differences between HbO changes following speech forward and speech backward stimuli across all channels and both hemispheres were correlated with the GA at birth using Pearson correlation and two‐tailed significance levels. We furthermore correlated both GA at birth and deviation from age appropriate weight at birth with speech discrimination abilities. The significance threshold was set to *p* ≤ 0.05.

#### 2.5.2 Hierarchical cluster analysis

Next, we applied an agglomerative hierarchical clustering technique to group patients according to GA at birth and discriminative abilities. Ward's method (minimum variance method) was used to combine pairs of clusters at each step. It starts with each single subject being one cluster and continues until all clusters are combined into a single cluster. Each new step is reached by minimizing variance, respectively the sum of square index. All of the 64 neonates were placed in their own cluster and then progressively clustered with others according to their GA and difference in HbO changes related to discriminative abilities. Discriminative abilities were again quantified by using the difference between mean HbO changes related to speech forward versus speech backward. To evaluate the relation between GA at birth and speech discrimination abilities at term‐equivalent age, we calculated correlation analyses within each subgroup and ran a 2 × 2 × 2 repeated measures ANOVA with the factors *Condition* (levels: forward and backward speech), *Hemisphere* (levels: left and right hemisphere), and *Group* to evaluate general differences. Analyses were carried out with IBM SPSS Statistics 19 software (IBM Corp., Armonk, NY, USA).

### 2.6 Nonparametric cluster‐based permutation test

Finally, we examined whether neonates responded differently to the two speech conditions over the whole time interval of 30 s post‐stimulus and each individual channel. To overcome the multiple comparison problems, we performed a cluster‐based permutation on the hemodynamic response of the HbO signal. The cluster‐based permutation approach is based on the assumption that effects associated with the different conditions are clustered along the dimensions of time and space (channels), thus it is possible to overcome the multiple comparison problem and still be able to include each time point sampled at each channel (Sassenhagen & Draschkow, 2019). First, we ran individual *t*‐tests between speech forward and speech backward condition for each pair of channels and for each time point (sampling rate was 10 Hz), separately for each hemisphere. Samples (every channel/time pair) were considered temporally adjacent when they were consecutive; channels´ spatial neighborhood was defined as channels within 2 cm distance from one another. The *t*‐score threshold for the cluster was +/−2.36 (which corresponds to a conventional alpha = 0.05 *p*‐value). All samples whose *t*‐score exceeded this threshold were selected. Two pairs of samples were clustered when exceeding this predefined threshold and when they were temporally consecutive and spatial adjacent. Next, cluster‐level *t*‐values were calculated by summing the *t*‐value of every data point included in the cluster. Clusters with a maximum *t*‐value were then taken and a permutation test was used to calculate whether this cluster belonged significantly to one condition and not to the other. To test whether the null hypothesis is true (i.e., the condition has no effect on the cluster), a total of 1000 permutations were conducted. To confirm the patterns observed in the cluster‐based permutation analyses, we tested whether HbO changes within these ROIs differed significantly between conditions using paired sampled *t*‐test.

## 3 RESULTS

### 3.1 Correlation analysis within the whole sample

**FIGURE 1:**
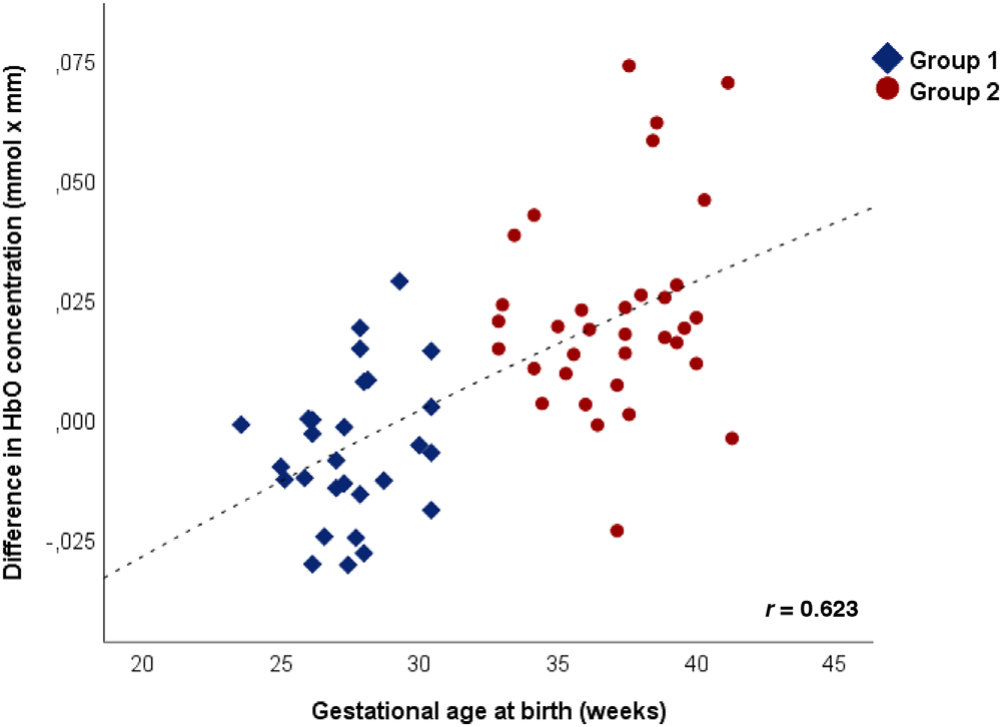
Difference of mean oxyhemoglobin (HbO) concentration change between speech forward and speech backward as a function of weeks of gestational age at birth. Results of cluster analysis are indicated by blue squares (group 1; born between week 23 and 31 of GA) and red dots (group 2; week 32 and 41 of GA)

Correlation analysis within the whole study sample revealed a significant positive correlation between GA at birth and differences in HbO changes between forward and backward speech across both hemispheres (*r* = 0.521, *p* < 0.001) at term‐equivalent age (Figure 1). Consistent with the high correlation of GA and weight at birth (*r* = 0.950; *p* < 0.001), birth weight also correlated with discriminative abilities (*r* = 0.539, *p* < 0.001). In contrast, the individual deviation from age appropriate weight at birth was not related to discriminative abilities (*r* = −0.105, *p* = 0.409).

### 3.2 Hierarchical cluster analysis

Hierarchical cluster analysis within the whole sample of study participants revealed two distinctive patterns of discriminative responses, thus dividing the infants into two groups. Subsequent two‐sample *t*‐tests showed that the groups not only significantly differed with respect to their discriminative abilities, but also disclosed significantly different GA at birth (*t* = −17.021, *p* < 0.001) and associated birth weight (*t* = −12.887; *p* < 0.001). Whereas infants in group 1 were born between week 23 and 31 of GA (mean = 27.63, SD = 1.75), infants in group 2 were born between week 32 and 41 of GA (mean = 37.07, SD = 2.42). In contrast, the groups did not differ with respect to their GA at the time of measurement (*t* = −0.684, *p* = 0.497) and their deviation from age appropriate birth weight (*t* = −0.693; *p* = 0.491). The supplementary file gives detailed information on clinical characteristics of subgroups.

**FIGURE 2:**
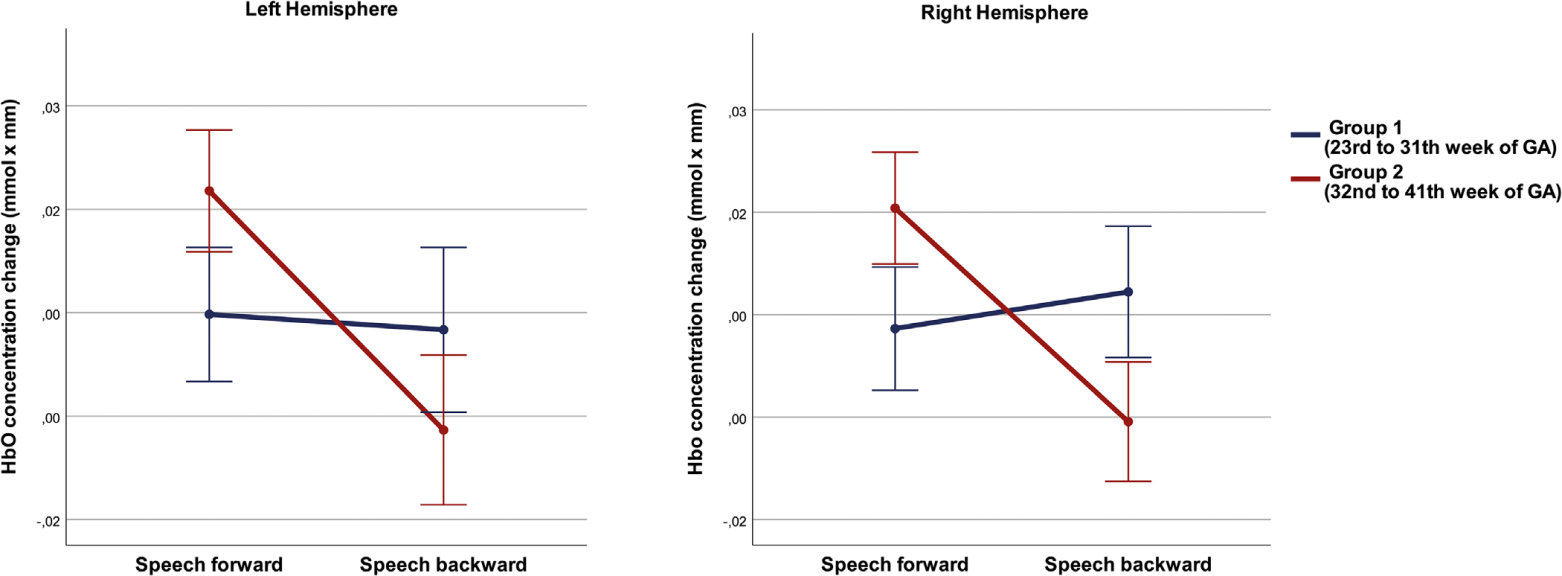
Interaction graphs (estimated marginal means) depicting the interaction effect between condition and group for each hemisphere. Error bars denote the standard error (+/−2)

Repeated measures ANOVA revealed a significant main effect on HbO concentration changes for the factor *Condition* (*F*(1,60) = 16.434, *p* < 0.001, η^2^ *p* = 0.215), but no significant difference between hemispheres (*F*(1,60) = 0.187, *p* = 0.667, η^2^ *p* = 0.003; Figure 2). In addition, we found a significant interaction effect for the factors *Condition* × *Group* (*F*(1,60) = 19.878, *p* < 0.001, η^2^ *p* = 0.249). Thus, the groups significantly differed with respect to their discriminative abilities (*t* = −4.809, *p* < 0.001). Group 1 (*n* = 28, 45.2% of study participants) exhibited a higher peak amplitude for HbO in the backward condition (0.006 +/− 0.01) compared to the forward condition (0.003 +/− 0.01) across all channels and both hemispheres (Figure 2). However, this difference between conditions in group 1 did not reach significance (*t* = −0.269, *p* = 0.790). Group 2 (*n* = 34; 54.8% of study participants) showed the opposite pattern, with a significantly higher peak amplitude in the forward condition (0.016 +/− 0.01) compared to the backward condition (−0.006 +/−0.02; *t* = 6.069, *p* < 0.001).

In sum, the analysis identified two subgroups of participants, with infants born before week 32 of GA showing a significantly different pattern of hemodynamic response to speech stimuli compared to infants born at or after week 32 of GA.

#### 3.2.1 Subsequent within‐groups analyses

**FIGURE 3:**
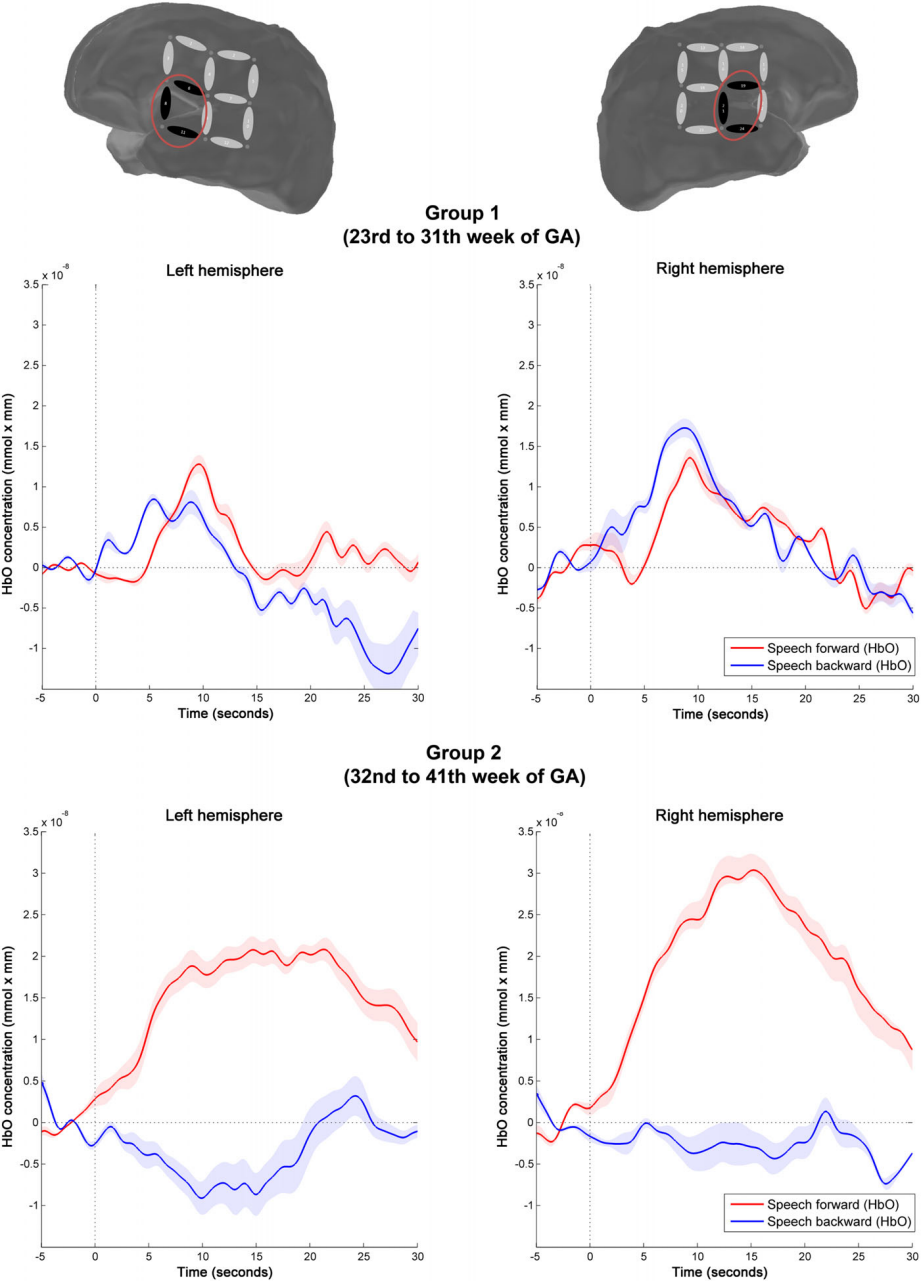
Mean time course of hemodynamic response to speech forward (solid line) and speech backward (dotted line) for group 1 (above) and group 2 (bottom). Red (HbO) and blue (HbR) shades indicate the standard deviation. The x‐axes display time in seconds, the y‐axes represent concentration changes in μmol/L, averaged over all channels and all stimuli per condition

Figure 3 shows the mean time course of hemodynamic response to speech forward and speech backward for each group. Analyses within each of the two groups revealed no significant relation between GA at birth and differences in HbO changes between forward and backward speech across both hemispheres, neither in group 1 (*r* = 0.236, *p* > 0.227) nor in group 2 (*r* = 0.185, *p* = 0.296). Likewise, within groups, neither actual birth weight (group 1: *r* = 0.227, group 2: *r* = 0.246) nor the deviation from age appropriate birth weight (group 1: *r* = −0.009, group 2: *r* = −0.233) correlated with differences in HbO changes between forward and backward speech (all *p* > 0.05).

### 3.3 Nonparametric cluster‐based permutation test

We furthermore performed a nonparametric cluster‐based permutation analysis investigating hemodynamic responses over the epoch of 0–30 ms post‐stimuli per group. This analysis of differences in HbO responses to forward compared to backward speech did not yield any significant cluster in group 1. In contrast, group 2 exhibited a significant difference between the speech forward and the speech backward conditions in two clusters. These clusters covered the anterior temporal lobes of each hemisphere, comprising channels 6, 8, and 11 on the left hemisphere (*t* = 3.272, *p* = 0.003) and channels 19, 21, and 24 on the right hemisphere (*t* = 5.008, *p* < 0.001), respectively. Within these two clusters, HbO differences started around 5 s after stimulus onset and remained throughout the whole epoch. Figure 3 displays the significant clusters in group 2, and for visual comparison, nonsignificant hemodynamic responses of group 1 within the same clusters (left cluster *t* = 0.241, *p* = 0.832, right cluster *t* = −0.259, *p* = 0.798).

When HbO responses to conditions were analyzed separately, group 1 exhibited a significant change of activation related to the speech backward condition in the right cluster (*t* = 2.275, *p* = 0.030), but no significant HbO change to speech forward in any of the clusters. In group 2, a significant change of activation related to the speech forward condition was observed both in the left (*t* = 5.492, *p* < 0.001) and in the right cluster (*t* = 3.868, *p* = 0.001), whereas no change in activation was found in response to the speech backward condition (all *p* > 0.05).

## 4 DISCUSSION

The present study investigated the impact of GA at birth on discrimination of speech prosody at term‐equivalent age within a large sample of neonates born between week 23 and 41 of GA. Correlation analysis revealed a positive relationship between GA at birth and speech discrimination at term‐equivalent age, and cluster analysis of HbO responses identified two subgroups of participants that differed in their age at birth: infants born before week 32 of GA exhibited a significantly different pattern of hemodynamic response to speech stimuli compared to infants born at or after week 32 of GA. Whereas the latter group showed significant speech discrimination in two neural clusters, the group born before week 32 of GA did not significantly discriminate forward from backward speech. This study not only proves a relationship between GA at birth and neural speech discrimination at term‐equivalent age; it furthermore suggests a critical threshold of around week 32 of GA for the intrauterine auditory cortex development.

The impact of GA at birth on discrimination of speech prosody at term‐equivalent age found in the present study underlines the importance of auditory cortex development in utero as the basis for early speech discrimination. Our findings of altered speech discrimination in preterm infants are in line with previous studies. Naoi et al. (2013) showed that at term‐equivalent age, preterm infants revealed decreased activity in response to speech stimuli in the right temporal region, but exhibited increased interhemispheric connectivity compared to full‐term infants. In a recent study of our own research group (Bartha‐Doering et al., 2019), we reported the absence of neural speech discrimination in a group of preterm infants at term‐equivalent age and a significant difference in HbO responses in preterm compared to full‐term infants. Both studies did not detect a correlation between GA at birth and speech discrimination; however, the sample sizes of the preterm groups were rather small (37 in Naoi et al.’s study, 15 in our previous study), and GA at birth was not well balanced either. In a further study, Arimitsu et al. (2018) described atypical patterns of phonetic and prosodic discrimination in neonates born between week 30 and 35 of GA. In this large study group of 80 neonates, preterm infants were not tested at term‐equivalent age but compared at different postnatal ages; however, a positive correlation between GA at examination and speech discrimination was found.

Correlation analyses between GA at birth and HbO responses indicate that the earlier the preterm birth, the higher the risk for speech discrimination deficits at term‐equivalent age, suggesting a linear increase across the whole study sample. However, cluster analysis of HbO differs between conditions identified two groups of infants with quantitative and qualitative differences in neuronal activations to speech stimuli. This analysis further identified week 32 of GA as a critical threshold: infants born before week 32 of GA did not differentiate between forward and backward speech, while infants born at or after week 32 of GA showed a significant difference between HbO concentration changes to forward versus backward speech. Whereas significant between‐group differences were found, within‐group results on speech discrimination did not significantly depend on GA at birth. Studies with larger sample sizes will have to clarify whether the lack of correlation of these within‐groups between GA at birth and speech discrimination is simply due to a lack of statistical power or indicates rather homogeneous groups in this regard. Either way, our results show that neonates born before week 32 of GA (generally defined as born very preterm) are at particular risk of speech discrimination deficits.

Previous studies have already underlined the time of around 32 weeks of GA as an important period in auditory cortex development. Evoked potential studies have shown almost mature biomechanical function of the cochlear signal at week 33 of GA (Morlet et al., 1995; Pasman et al., 1991), whereas prior to this age, the immature auditory pathways cannot relay the information from the periphery to the cortex (Jardri et al., 2008). As a consequence, several studies suggest the beginning of the development of memory traces starts around week 32 of GA: Decasper et al. (1994) have investigated the heart rates of fetuses during recitals of children's rhymes by their mothers and found specific heart rates in response to stimulation with previously heard rhymes starting around the GA of 32 weeks (Decasper et al., 1994). Similarly, Morokuma et al. (2004) observed habituation in fetuses to repeated sound stimuli from week 32 of GA on. Jardri et al. (2008) have furthermore used functional magnetic resonance imaging to investigate brain activation to sound in fetuses between the week 28 and 34 of GA and found specific activations to sound in the left temporal cortex in fetuses with week 33 of GA and older. These findings together with ours suggest a significant change in processing of complex speech sounds around week 32 of GA.

A previous study with premature human infants investigating syllable discrimination suggests that infants born before week 32 of GA are able to distinguish a place of articulation contrast (e.g., ba/ga) (Mahmoudzadeh et al., 2013). Here, we extend the analysis to whole strings of continuous speech, and found that infants born before week 32 of GA showed impaired speech discrimination capacities (backwards vs. forwards) at term‐equivalent age compared to infants born between week 32 and 41 of gestation. Therefore, considering that even very preterm infants are capable of discriminating between phonemes (Mahmoudzadeh et al., 2013) and that many segments sound alike in both forward and backward speech (Dehaene‐Lambertz et al., 2002), it seems reasonable to infer that infants’ speech discrimination capacity (when present) may not rely on phonemic distinctions per se but on other cues that are distorted when the signal is played backwards.

We argue that newborns might be responding to prosodic cues. Several observations are consistent with this hypothesis. First, at least part of the prosodic information carried by vowels is available to the fetus in the womb. Surrounded by maternal tissues, amniotic fluid, and maternal noises including heartbeat, respiration, and intestinal activity, the fetus predominantly experiences low‐frequency sounds (Gerhardt & Abrams, 2000). Second, neonates’ auditory memory traces rely more on vowels (Benavides‐Varela et al., 2012) that, crucially, are a rich source of information about the prosodic structure of language. Third, behavioral studies have demonstrated that neonates can discriminate between a pair of languages differing in their rhythmical properties only when the sentences are played forwards and not when the utterances are reversed (Ramus et al., 2000), suggesting that rhythmical/prosodic properties are crucial for supporting the neonate's discrimination capacities. Fourth, prosody appears to be a strong cue for the identification of linguistic units at birth (Benavides‐Varela & Gervain, 2017).

In light of the above evidence, it might be hypothesized that preterm birth impacts this early sensitivity to prosodic information. Previous studies have shown that auditory cortex maturation is disrupted by preterm birth (McMahon et al., 2012). At term‐equivalent age, white matter properties of the auditory cortices have been shown to be significantly different in preterm born infants compared to full‐term born neonates, reflecting either delayed maturity or injury (Monson et al., 2018). Furthermore, preterm neonates spend their first weeks of life in the neonatal care unit, some of them several weeks within the incubator. During this time, they are deprived of the biological maternal sounds they would have heard inside the womb, including the low‐frequency bands of voices, maternal heartbeat, or digestion. Moreover, children nursed within an incubator are exposed to high levels of noise, caused by air supply systems, which hinder them from perceiving speech sounds from outside the incubator (Bertsch et al., 2020). Deprivation of maternal sounds together with environmental noise increase may have an additional negative effect on auditory brain maturation and subsequent speech and language acquisition. These three factors combined (disruption of structural auditory cortex maturation by preterm birth, deprivation of biological sounds, and noise increase) probably add to the negative impact of preterm birth on early language sensitivity, with additional complications when disruption occurs before the immature auditory pathways have learned to relay the information from the periphery to the cortex, which happens around week 32 of GA (Jardri et al., 2008).

The speech discrimination deficits observed in the present study may just be a sign of delayed developmental trajectory. It may be that preterm infants simply need more time to learn prosodic features and that they might catch up later in childhood. Preterm infants might also use different strategies to acquire language (Saffran & Thiessen, 2003). However, previous studies on linguistic development have shown that preterm born children often display prosodic discrimination deficits early in childhood (Herold et al., 2008). Impaired discrimination of speech rhythm and of changes in the amplitude of speech sounds is associated with developmental language disorders (Goswami, 2019). Reduced stress pattern discrimination in 5‐month‐old infants is a marker of risk for later language impairment (Weber et al., 2004). Furthermore, the ability to process phrasal prosody impacts learning of important aspects of language also later in development, including the organization of information in conversation, word segmentation, and syntactic parsing (Prieto & Esteve‐Gibert, 2018; Speer & Ito, 2009). However, an association between neural speech prosodic discrimination at birth and later language abilities in preterm born children has not yet been proven. Thus, further research is needed to illuminate if prosodic discrimination at birth maybe serves as a biomarker of risk of a later language developmental deficit.

### 4.1 Limitations

Although all infants were tested around week 38 of GA, the group of infants born between week 23 and 31 of GA showed a smaller amplitude of HbO concentration changes overall, irrespective of condition, compared to the group of infants born between week 32 and 41 of GA. These magnitude differences point to differences between groups in the neurovascular coupling per se (Nourhashemi et al., 2019). To overcome this issue, the present study measured relative intra‐subject differences of HBO concentration changes to forward versus backward speech stimuli. The groups not only differ with regard to relative differences to stimuli, but also showed distinctive patterns of discriminative responses. Nevertheless, the present study only used auditory stimuli and thus cannot clarify if the findings are specific to auditory discrimination or reflect an overall discrimination deficit as early as with 40 weeks of GA.

Previous studies have shown an increased risk of neurological impairment in preterm born infants (du Plessis, 2009), and 2%–4% of preterm born neonates suffer from congenital or perinatal hearing loss (Coenraad et al., 2011; Colella‐Santos et al., 2014; Kang et al., 2012). The present study, however, included only neonates with a normal auditory brainstem response and normal neurological findings. While we believe that these inclusion criteria are important to reduce the groups’ heterogeneity, these strict criteria have limited the generalizability of findings to the whole population of preterm born infants.

### 4.2 Conclusions

This study emphasizes the risk of speech discrimination deficits in infants born preterm, with a significant correlation between duration of intrauterine development and neural discrimination of speech prosody at term‐equivalent age, and identifies a critical threshold of auditory cortex development around week 32 of GA. Children born before this age are thus especially vulnerable to early speech discrimination deficits, which may in turn result in language developmental delays. We therefore suggest a close follow‐up and additional speech and language therapy, especially in the group of children born before this critical threshold, in order to support their early language development.

## CONFLICT OF INTEREST

The authors declare no competing interests.

## Supporting information

Supporting Information

## Data Availability

The data that support the findings of this study are available from the corresponding author upon reasonable request.
