# Supplementary material for: The duration of intrauterine development influences discrimination of speech prosody in infants
Source: Dev Sci. 2021 Apr 4;24(5):e13110. doi: 10.1111/desc.13110 (PMC11475226; doi:10.1111/desc.13110)
Supplement: Supplementary file 1 — Supporting Information [file DESC-24-e13110-s001.docx]

**Supplementary Material**

# Clinical data

Supplementary Table 1. Perinatal Characteristics and frequency of assisted ventilation among infants

|  | **Group 1 (n=28)** | **Group 2 (n=34)** |
| --- | --- | --- |
| **Perinatal characteristic** |  |  |
| Female, n (%) | 18 (64.3) | 15 (44.1) |
| GA at birth, weeks, mean (SD) | 27.63 (1.75) | 37.07 (2.42) |
| HC at birth, mm, mean (SD) | 25.27 (2.46) | 33.47 (1.94) |
| Birth weight, g, mean (SD) | 1008.00 (280.83) | 2747.35 (713.30) |
| Birth height, mm, mean (SD) | 35.42 (3.63) | 48.40 (3.99) |
| Apgar score at 1 min, median, range | 8 (6-9) | 9 (7-10) |
| Apgar score at 5 min, median, range | 9 (8-9) | 10 (8-10) |
| Apgar score at 10 min, median, range | 9 (9-9) | 10 (9-10) |
| Antenatal glucocorticoids, N (%) | 26 (92.9) | 2 (5.8) |
| Exogenous sufactant, N (%) | 18 (64.3) | - |
| Duraton of stay | 51.00 (32.0) | 2.45 (0.51) |
| **Assisted ventilation or oxygen** |  |  |
| Nasal IMV or SIMV, N (%) | 10 (35.7) | - |
| High flow nasal cannula, N (%) | 26 (92.9) | 1 (3.0) |
| High frequency ventialtion, N (%) | 1 (3.6) | - |
| convential ventilation, N (%) | 7 (25.0) | 1 (3.0) |
| nasal CPAP, N (%) | 27 (96.4) | 1 (3.0) |
| Oxygen, N (%) | 26 (92.9) | 2 (5.8) |
| Oxygen at 36 weeks, N (%) | 5 (17.9) | - |

Note: CPAP = continuous positive airway pressure; g = gram; GA = gestational age; HC = head circumference; IMV = intermittent mandatory ventilation; min = minutes; N = number; SIMV = synchronized intermittent mandatory ventilation

# Results of HbR concentration changes

**Correlation analysis within the whole sample**

Correlation analysis within the whole study sample revealed a significant positive correlation between GA at birth and differences in HbR changes between forward and backward speech across both hemispheres (r = -0.253, p = 0.048).

**Hierachical cluster analysis**

ANOVA revealed that mean HbR changes differed significantly between hemispheres (F(1,61) = 7.509, p = 0.008, η2p = 0.110), but not between conditions (F(1,61) = 0.469, p = 0.496, η2p = 0.008). Across groups and conditions ANOVA revealed a significantly less negative deflection of HbR concentration changes in the left compared to the right hemisphere. No interaction effect between any of the three factors *(Condition, Hemisphere*, and *Group*) was found (for all p > 1.0).

**Subsequent groups analyses**

Correlation analyses within each of the two groups revealed no significant relation between GA at birth and differences in HbR changes between forward and backward speech across both hemispheres, neither in group 1 (r = 0.070, p = 0.722) nor in group 2 (r = -0.319, p > 0.066). Likewise, within groups, neither actual birth weight (group 1: r = -0.147, group 2: r = -0.182) nor the deviation from age appropriate birth weight (group 1: r = -0.322, group 2: r = -0.106) correlated with differences in HbR changes between forward and backward speech (all p > 0.05).

**Nonparametric cluster‐based permutation**

Nonparametric cluster-based permutation on the hemodynamic response of the HbR signal did not yield any significant cluster in group 1 nor in group 2.
